# Do Gender-Predominant Primary Health Care Organizations Have an Impact on Patient Experience of Care, Use of Services, and Unmet Needs?

**DOI:** 10.1177/0046958017709688

**Published:** 2017-06-04

**Authors:** Raynald Pineault, Roxane Borgès Da Silva, Sylvie Provost, Michel Fournier, Alexandre Prud’homme, Jean-Frédéric Levesque

**Affiliations:** 1 Institut National de Santé Publique du Québec, Montréal, QC, Canada; 2 Centre de Recherche du Centre hospitalier de l’Université de Montréal, Montréal, QC, Canada; 3 Institut de Recherche en Santé Publique de l’Université de Montréal, Montréal, QC, Canada; 4 Faculté des Sciences Infirmières de l’Université de Montréal, Montréal, QC, Canada; 5 Direction de Santé Publique du Centre Intégré Universitaire de Santé et de Services Sociaux du Centre-Sud-de-l’Île-de-Montréal, Montréal, QC, Canada; 6 Bureau of Health Information of New South Wales, Australia; 7 University of New South Wales, Sydney, Australia

**Keywords:** primary health care, gender-predominant organizations, feminization of medicine, experience of care, use of services

## Abstract

Physicians’ gender can have an impact on many aspects of patient experience of care. Organization processes through which the influence of gender is exerted have not been fully explored. The aim of this article is to compare primary health care (PHC) organizations in which female or male doctors are predominant regarding organization and patient characteristics, and to assess their influence on experience of care, preventive care delivery, use of services, and unmet needs. In 2010, we conducted surveys of a population stratified sample (N = 9180) and of all PHC organizations (N = 606) in 2 regions of the province of Québec, Canada. Patient and organization variables were entered sequentially into multilevel regression analyses to measure the impact of gender predominance. Female-predominant organizations had younger doctors and nurses with more expanded role; they collaborated more with other PHC practices, used more tools for prevention, and allotted more time to patient visits. However, doctors spent fewer hours a week at the practice in female-predominant organizations. Patients of these organizations reported lower accessibility. Conversely, they reported better comprehensiveness, responsiveness, counseling, and screening, but these effects were mainly attributable to doctors’ younger age. Their reporting unmet needs and emergency department attendance tended to decrease when controlling for patient and organization variables other than doctors’ age. Except for accessibility, female-predominant PHC organizations are comparable with their male counterparts. Mean age of doctors was an important confounding variable that mitigated differences, whereas other organization variables enhanced them. These findings deserve consideration to better understand and assess the impacts of the growing number of female-predominant PHC organizations on the health care system.

## Introduction

The increasing number of women in medicine is a phenomenon that has been observed in many countries.^1,2^ In 2000, women represented about 30% of all Canadian physicians. In 2017, this percentage was up to 41% and varied among Canadian provinces from 32.4% to 48.0%. Québec had the highest percentage (48.0%).^3^

In Canada, primary health care (PHC) physicians are predominantly paid on a fee-for-service basis although blended modes of remuneration that include capitation are emerging.^4-6^ While nearly all doctors are reimbursed through public health insurance programs, most PHC practices are privately owned by physicians. Solo practice remains an important PHC organization, representing about 30% of all PHC organizations, but is a declining mode of PHC services delivery.^7,8^

Female physicians are different from their male counterparts in many ways. They are more likely to choose family medicine, pediatrics, psychiatry, and obstetrics-gynecology.^9^ They also have a different style of practice: They spend more time with patients, provide more preventive services, work fewer hours per week, and engage more in patient-centered communication.^10-17^ The bulk of research that has looked at differences between female and male physicians has focused on individual characteristics of physicians rather than organizations in which they carry out their clinical activities. To our knowledge, no study has ever attempted to determine the extent to which gender predominance as an organizational characteristic can influence medical practice in PHC organizations. Gender predominance in an organization refers to which of the 2 genders is more influential and is generally expressed by percentages of genders among its members.

The hypothesis of the influence of gender predominance in health care organizations is based on the belief that while physicians’ individual characteristics may establish criteria for joining an organization, they do not fully explain actual clinical behaviors of physicians.^17-18^ Every work setting has its own source of influence over the work of individual physicians. This influence is mainly conveyed through professional values and norms prevailing in a given organization and shared by the majority of its professionals. Assuming that female physicians hold different values and norms regarding medical practice, gender predominance in an organization may constitute an important determinant of physicians’ style of practice in that organization.^12,13^

This is the issue we address in this article. Specifically, our objectives are 2-fold:

1. Compare female- and male-predominant types of PHC organizations with respect to patient and organization characteristics;
2. Assess the influence of gender-predominant PHC organizations on patients’ experience of care, preventive care delivery, use of services, and unmet needs.

## Methods

### Surveys: Sampling and Questionnaires

Our study is based on data gathered through 2 surveys carried out in 2010 in 2 densely populated regions of the province of Québec (Canada), which account for 43% of the total province’s population.^19,20^ Using the random-digit dialing method, we carried out a population-based telephone survey of 9180 adults aged 18 or older. The response rate for that survey was 56%. Data were weighted by attributing the inverse probability of participant selection associated with the 2-stage sampling procedure used: a nonproportionally area sample of about 400 respondents in each of the 23 areas and an intrahousehold random sample. We also applied a poststratification adjustment for age and sex based on 2010 estimated Canadian census data. The population-based questionnaire focused on patients’ assessment of attachment to a regular source of care, use of services, experience of care, unmet needs, and preventive care. Questions related to experience of care were inspired from 2 widely known instruments: the Primary Care Assessment Survey and the Primary care Assessment Tool.^21-22^ Data collected with the questionnaire used in our study have been reported in previous published articles.^7,23-25^

For the second survey, a mailed questionnaire was sent to all PHC organizations in the 2 regions under study (N = 606). The response rate was 62%. Prior to the mail survey, every organization was contacted by telephone and asked to identify a key informant, usually the physician in charge of professional and administrative matters. This person was subsequently asked to complete the questionnaire. We also took advantage of this telephone contact to obtain basic information about the organization such as number of physicians in the practice; whether there were nurses, specialists, or other health professionals in the same setting; and prevailing mode of visits (by appointment or walk-in). Based on this information, we applied a hot-deck imputation procedure for missing data, conditional on the geographical area, and type and size of each nonresponding PHC organization.^26,27^

The organization questionnaire included 4 dimensions that characterize any organization: Vision refers to values, norms, and goals shared by members of an organization; resources concern type and quantity of human, material, and financial resources available; structure refers to governance rules and procedures; and processes include administrative and professional mechanisms put in place to ensure adequate service delivery. Formulation of questions was informed by the literature and, more particularly, proposals of organizational models based on the Patient-Centered Medical Home concept.^28-30^ Issues related to validation of the questionnaire were addressed in a previous publication.^31^ Interestingly, the Canadian Institute for Health Information has included the major part of the questionnaire in its own questionnaire designed to be used by Canadian researchers involved in assessment of PHC organizations.^32^

Organization and population surveys were linked through population survey respondents’ identification of their usual source of primary care in the 2 years prior to the survey or, if not possible, of the practice they attended the most often. Information provided by respondents on name and location of practices and of their doctors was then compared with information available from the research team’s registry to do the matching. This practice was considered as their usual source of primary care. Of the 6885 respondents who had a regular source of care, only 403 could not be linked either because we did not get enough information from the respondent or because the respondent lived outside the 2 regions or the practice was closed.

For the purpose of this article, solo practices were not included in the analysis because they represent an extreme case of gender predominance (100%) and are very different from group practices on so many aspects that their inclusion generated more confusion than clarification. In addition, we wanted to focus on emerging forms of practice rather than on a declining one.^33,34^

The project has received ethical approval from the main research ethics committee of the Agence de la santé et des services sociaux de Montréal. The multicenter nature of the research project also required ethical approval from research ethics committees of the 23 health and social services centers participating in the study.

### Organization Variables

The main organization variable was gender-predominant types of PHC organization. The construction of this variable was based on the theoretical notion that group influence in the work place is most likely to be conveyed by those who constitute the majority of members and those in leadership positions. This is particularly the case in a professional organization where all doctors have an equal professional status. We thus used 2 indicators (percentage of female physicians and whether they occupy leadership positions), to construct the variable gender predominance. Simple gender majority was deemed to be a valid indicator of gender predominance. The variable was dichotomized into female- and male-predominant, based on the percentage (55% or more) of male or female doctors in the PHC organization. For the 45% to 55% category, sensitivity analyses showed that simple majority did not discriminate enough to properly classify organizations in either of the 2 gender-predominant groups. We decided to be more parsimonious and apply an additional criterion for that category, based on the gender of the doctor who was designated as the leader of the medical group. Organizations that had between 45% and 55% of male or female doctors were thus included in one of the 2 categories, depending on the gender of the medical leader. Given the logic that underpinned the construction of the variable gender predominance, a dichotomous variable was more appropriate than a categorical or a continuous scale. The final classification yielded 2 categories: female-predominant representing 44.2%, and male-predominant representing 55.2% of PHC organizations. The former was the usual source of primary care for 39.7% of population survey respondents who had a usual source of primary care and the latter for 60.3%. Details concerning operationalization of the other organizational variables used in the analyses are presented in Supplementary File 1.

### Dependent Variables

Dependent variables included experience of care and use of services in the 2 years preceding the survey (2008-2010) as reported by respondents to the population questionnaire. Also included were unmet needs in the 6 months prior to the survey and preventive care received according to clinical guideline specifications.^20,35^ Indicators of experience of care and preventive care were expressed on a 10-point scale.^24^ We carried out a factor analysis for each of the 5 dimensions of patient experience of care; we also calculated Cronbach alpha that reached values of 0.60 or more for continuity (0.61), comprehensiveness (0.79), responsiveness (0.63), and outcomes (0.82), but was lower in the case of accessibility (0.30), presumably because that scale was more formative than reflective.^36,37^ Details concerning operationalization of the dependent variables are presented in Supplementary File 2.

### Data Analysis

We first compared the 2 types of PHC organization for patient and organization characteristics using the chi-square test with the adjusted standardized residuals procedures to identify significant differences between groups.^38^ Size of practice measured by number of physicians was presented as a descriptive indicator in contrasting female- and male-predominant PHC organizations, but was left aside in regression analyses because it correlated highly with most organization variables.^24^

To measure associations between gender-predominant PHC organizations and the dependent variables, we applied multilevel linear or logistic regression, depending on whether the outcome variable was continuous or categorical. In the regression analysis models, we controlled for patient characteristics (level 1) and organization characteristics (level 2) to determine the effects of gender predominance types. The impact of sets of independent variables was assessed by entering sequentially in our models mean age of doctors in the practice, patient characteristics, and other organization variables. Statistical analyses were performed using Stata SE 13.

## Results

### Organization Characteristics

As shown in Table 1, female- and male-predominant PHC organizations differ significantly on many characteristics. Doctors in female-predominant organizations tend to be younger. These organizations have also more often a nurse who provides follow-up for patients with chronic diseases and counseling. Their collaborative links with other PHC practices are better established. They also allow patients longer time for visits and have set up more mechanisms for maintaining competency. However, the involvement of doctors in female-predominant organizations, measured by the average number of hours spent weekly in the practice setting, is lower than in male-predominant organizations.

**Table 1.** Differences in Organizational Characteristics (%) Between Gender-Predominant Types of PHC Organization.

	Gender-predominant		Total (n = 393)	*P* ^a^
	Male (n = 217)	Female (n = 176)		
	%	%	%	
Number of physicians in the practice				
2 physicians	20.2	18.2	19.4	.176
3-5 physicians	37.8	29.0	33.8	
6-9 physicians	22.2	27.2	24.4	
10 physicians or more	19.8	25.6	22.4	
Average age of physicians in the practice				
30-48	18.0 (−)	56.8 (+)	35.4	<.001
49-56	35.5 (+)	25.0 (−)	30.8	
57-59	25.3 (+)	10.8 (−)	18.8	
60 or more	21.2 (+)	7.4 (−)	15.0	
Time devoted by nurses to follow-up of patients with chronic diseases				
No	64.1	51.1	58.3	.010
Yes	35.9 (−)	48.9 (+)	41.7	
Time devoted by nurses to counseling on healthy habits				
No	67.3	51.7	58.3	.002
Yes	32.7 (−)	48.3 (+)	41.7	
Use of at least one information technology				
No	24.4	18.2	20.4	.135
Yes	75.6	81.8	79.6	
Collaboration with other PHC practices				
No	65.0	51.7	59.0	.008
Yes	35.0 (−)	48.3 (+)	41.0	
Collaboration with hospitals				
No	47.0	44.9	46.1	.675
Yes	53.0	55.1	53.9	
Services offered on evenings or weekends				
No	37.3	35.8	36.6	.754
Yes	62.7	64.2	63.4	
Prevailing type of visits in the practice				
Walk-in	18.0	12.5	15.5	.244
By appointment	52.1	59.1	55.2	
Mixed	29.9	28.4	29.3	
Length of time allowed for each visit				
Shortest	40.6 (+)	27.3 (−)	34.6	.001
Moderate	29.0	24.4	27.0	
Longest	30.4 (−)	48.3 (+)	38.4	
Range of diagnostic or therapeutic services available				
Lowest	6.0	6.3	6.1	.770
Moderate	70.5	73.3	71.8	
Highest	23.5	20.4	22.1	
Number of tools and mechanisms available for preventive services delivery				
Lowest	15.7	14.8	15.3	.120
Moderate	54.8	46.0	50.9	
Highest	29.5 (−)	39.2 (+)	33.8	
Number of mechanisms in place for maintaining competency				
Lowest	23.0	18.2	20.9	.002
Moderate	56.2	48.9	52.9	
Highest	20.7 (−)	32.9 (+)	26.2	
Average number of hours physicians devote weekly to clinical activities in the setting				
Less	30.0	36.9	33.1	.001
Moderate	38.2	47.7	42.5	
More	31.8 (+)	15.4 (−)	24.4	

*Note.* Standardized Pearson residual larger (+) than 2 or smaller (−) than −2. PHC = primary health care.

a Chi-square test.

### Patient Characteristics

As shown in Table 2, female-predominant PHC practices have higher percentages of female patients, individuals aged 30 to 44 and with higher education and economic status. For all other variables, there is no significant difference between the 2 types of PHC organizations.

**Table 2.** Differences in Patient Characteristics (%) Between Gender-Predominant Types of PHC Organization.

	Gender-predominant		Total (n = 6084)	*P* ^a^
	Male (n = 3669)	Female (n = 2415)		
	%	%	%	
Gender (patient)				
Male	47.9	40.5	45.0	<.001
Female	52.1 (−)	59.5 (+)	55.0	
Age (patient)				
18-29	18.0	16.5	17.4	.075
30-44	26.1 (−)	29.0 (+)	27.2	
45-64	36.6	36.1	36.4	
65 or more	19.3	18.4	19.0	
Level of education				
Primary	12.7	11.9	12.3	.015
Secondary	29.6	28.6	29.2	
College	22.5 (+)	20.4 (−)	21.7	
University	35.2 (−)	39.1 (+)	36.8	
Economic status				
Lowest	9.0 (+)	7.5 (−)	8.4	.003
Mid-lower	30.0	28.0	29.2	
Mid-higher	35.0	34.4	34.7	
Highest	26.0 (−)	30.1 (+)	27.7	
Perceived health status				
Bad or average	15.4	14.1	14.9	.468
Good	30.4	30.2	30.3	
Very good	34.4	35.9	35.0	
Excellent	19.8	19.8	19.8	
Have a chronic disease^b^				
No	41.7	40.8	41.3	.500
Yes	58.3	59.2	58.7	

*Note.* Standardized Pearson residual larger (+) than 2 or smaller (−) than −2. PHC = primary health care.

a Chi-square test.

b Includes coronary artery disease, heart failure, chronic obstructive pulmonary disease, asthma, arthritis, osteoarthritis, rheumatism, hypertension, diabetes, hypercholesterolemia, cancer, HIV, anemia, and gastrointestinal disorders.

### Effect of Gender-Predominant PHC Organizations on Patient Experience of Care, Preventive Services, Unmet Needs, and Use of Services

Tables 3 and 4 present the results of 4 regression analysis models for all dependent variables, with cumulative sequential entries of different sets of independent variables: gender-predominant PHC organization (model 1) to which doctors’ mean age was added (model 2), then patient characteristics (model 3), and finally a model that includes all organizational characteristics (model 4). Table 3 presents the results of linear regressions for continuous dependent variables. Of these variables, only accessibility remains significant throughout the 4 steps, indicating a direct negative impact of female-predominant PHC organizations. Comprehensiveness, responsiveness, counseling, and screening practices show positive associations with female-predominant PHC organizations in the unadjusted model, but this association fades when doctors’ mean age is entered in the model (model 2) and becomes even less significant when patient and other organization variables are added in regression analyses (models 3 and 4). This suggests that the effect of female organizations on these outcome variables is confounded with that of doctors’ mean age in the practice setting. For screening, the positive association decreases with model 2 and vanishes completely with model 3, suggesting confounding effects of both doctors’ age and patients’ characteristics.

**Table 3.** Association of Gender-Predominant Types of PHC Organization With Patient Experience of Care and Preventive Services Delivery (Mean Score on 10-Point Scales).

	Gender-predominant (Ref.: Male)							
	Model 1^a^		Model 2^b^		Model 3^c^		Model 4^d^	
	Female		Female		Female		Female	
	β	*P*	β	*P*	β	*P*	β	*P*
Experience of care								
Accessibility of services	−.224	.006	−.279	.003	−.241	.008	−.212	.022
Continuity of care	−.002	.998	.143	.197	.117	.217	.030	.746
Comprehensiveness	.227	.022	.137	.215	.139	.181	.028	.793
Responsiveness	.123	.039	.056	.376	.073	.234	.029	.654
Perceived outcomes	.055	.533	.013	.893	.016	.861	−.086	.342
Preventive services								
Lifestyle habits counseling	.301	.042	.169	.299	.184	.247	.011	.949
Cancer and cardiometabolic disorders screening	.345	.021	.270	.094	.209	.169	.155	.334

*Note.* PHC = primary health care.

a Unadjusted.

b Adjusted for doctors’ age.

c Adjusted for doctors’ age and patient characteristics.

d Adjusted for doctors’ age, patient characteristics, and organizational characteristics.

**Table 4.** Association of Gender-Predominant Types of PHC Organization With Patients’ Unmet Needs and Use of Services.

	Gender-predominant (Ref.: Male)							
	Model 1^a^		Model 2^b^		Model 3^c^		Model 4^d^	
	Female		Female		Female		Female	
	OR	*P*	OR	*P*	OR	*P*	OR	*P*
Unmet needs								
Did not consult doctor when needed	0.891	.142	0.876	.127	0.852	.056	0.848	.057
Use of services								
Hospitalization (≥1)	1.012	.864	0.971	.704	0.977	.764	0.986	.861
ED (≥1)	0.944	.361	0.918	.221	0.924	.254	0.869	.058
At the usual source of primary care (≥6)	0.913	.269	0.890	.195	0.889	.194	0.855	.092

*Note.* PHC = primary health care; OR = odds ratio; ED = emergency department.

a Unadjusted.

b Adjusted for doctors’ age.

c Adjusted for doctors’ age and patient characteristics.

d Adjusted for doctors’ age, patient characteristics, and organizational characteristics.

Table 4 shows the results of the logistic regression analyses for dichotomous dependent variables (unmet needs and use of services). Odds ratio (OR) for unmet needs decrease after patient and other organization characteristics are introduced in the models, but *P* values do not reach the .05 level of significance (models 3 and 4). The progression from model 1 to model 4 seems to suggest that patients attached to female-predominant PHC organizations are likely to report fewer unmet needs than patients with male-predominant organizations because the effect, measured by ORs, is enhanced after we adjust for patient and organization variables other than mean age of doctors.

The pattern for emergency department (ED) attendance and use of primary care services at the usual source of care is quite similar: OR values decrease when other organization characteristics (model 4) are added (Table 4), but *P* values never reach the .05 level of significance. Decreasing values of ORs suggest that organization variables other than mean age of doctors coupled with female-predominant PHC organizations would have more influence on use of ED and primary care services than gender-predominant PHC organizations alone.

To further determine which organization variables intervene to modify the relationship between gender-predominant PHC organizations and outcome variables, we carried out a change-in-estimate regression analysis that measured, for each outcome variable, the change in the gender-predominant coefficient (β) brought about by the introduction of each organizational variable (data not presented). Three variables emerged as major contributors of change for most dependent variables: range of therapeutic and diagnostic services available, length of time allowed for each visit, and average number of hours physicians devoted weekly to clinical activities in the practice.

## Discussion

Female-predominant PHC organizations differed from male-predominant ones in many aspects. They had younger doctors, more often employed nurses with expanded roles in follow-up of chronic diseases and in counseling, allowed more time for visits, and had more tools and mechanisms available to foster prevention and maintain competency; however, their doctors spent fewer hours a week at the practice. Profiles of patients attached to the 2 types of organizations were comparable, with the exception of a higher percentage of female patients and patients aged 30 to 44, and a slightly higher proportion of patients with higher education and economic status.

Patients attached to female-predominant PHC organizations reported lower accessibility to services. The effect of female-predominant PHC organizations remained unchanged after introducing organization and patient characteristics, indicating a direct effect on accessibility. Among the other indicators of experience of care, comprehensiveness, responsiveness, counseling and, to a lesser extent, screening were all associated positively with female-predominant PHC organizations, but their influence on patient experience of care was confounded with that of other organization variables, particularly mean age of doctors in the practice. These aspects of patient experience of care found to be positively associated with female-predominant PHC organizations before adjusting for confounders lost significance after adjustment. In sum, the positive influence of female-predominant organizations seemed to be more related to the younger age of doctors than to gender predominance.

The result for unmet needs indicates that patients attached to female-predominant PHC organizations were less likely to report unmet needs than patients of male-predominant organizations only after being adjusted for patient and organization characteristics other than age of doctors. Although the difference between the 2 types of organization never reached the .05 level of significance, the changes observed in the ORs with models 2 and 3 suggest that patient characteristics would have an enhancing effect on this outcome variable. For ED attendance and use of primary care services, organizational variables other than mean age of doctors seemed to mediate the positive association between female-predominant organizations and the 3 most contributing organizational variables: number of hours spent weekly in the practice, length of time planned for a visit, and range of diagnostic and therapeutic procedures offered.

One of the main results of our study is that patients attached to female-predominant PHC organizations reported lower accessibility of services compared with male-predominant ones, but this unfavorable result was not associated with other negative outcomes such as reporting more unmet needs or ED attendance and less use of primary care services. These indicators are generally correlated with accessibility. For example, higher ED attendance is possibly associated with increased use of services for conditions that can be avoided with appropriate use of ambulatory care services or that are amenable to prevention.

These results raise concerns about causal relationships between gender predominance and other organizational variables. A puzzling question is whether doctors select a PHC organization with particular characteristics or contribute to changing their organization after joining it. Our cross-sectional study does not enable us to respond to that question. Longitudinal studies are needed to disentangle these 2 effects. However, it is reasonable to believe that the influence of gender predominance on outcome variables probably results from the selection by female and male doctors of practice settings that correspond to their values and career plans as well as the pressures they exert to shape the host organization in accordance with their aspirations. It was impossible to separate out the specific contributions of female and male doctors because we did not have information on individual physicians.

A similar reasoning applies to the relationship between mean age of doctors, which we consider an organizational characteristic, and gender-predominant organizations. From an organizational perspective, the increased supply of female doctors in recent years has resulted in recruitment of younger doctors. Again, as we do not have information on individual characteristics of physicians, we cannot determine what the respective contributions of male and female doctors are on lowering the mean age of doctors in female-predominant organizations. All we can say is that recently recruited female and male doctors may have contributed to lower the mean age of doctors in female-predominant organizations. This effect is more likely due to female recruitment because of the enlarged pool of female doctors in family medicine. In brief, younger mean age of doctors in female-predominant organizations likely reflects both a selection effect and a more dynamic process of developing new innovative models of organization. In that sense, younger age of doctors in female-predominant organizations can be seen as a consequence of the process of recruiting doctors and developing a new form of organization in conformity with female doctors’ values and orientations.

Since our study focused on PHC organizations, it is difficult to compare our results with those of studies that have looked at individual characteristics of doctors in examining their different styles of practice. Nevertheless, our results concur with the findings of several of these studies regarding delivery of prevention services, length of time spent with patients, utilization of ED services and patient-physician relationship, all of which seem to favor female doctors.^11,15,39^ Our study clearly shows that female-predominant PHC organizations provide lower accessibility care, as perceived by their patients. This negative effect was also reported in a review by Hedden et al.^12^ Patients of female-predominant organizations did not rate other aspects of experience of care higher than patients of male-predominant ones, when adjusting for patient and other organization confounders. Conversely, some outcomes, as shown by changes in ORs, seemed to improve when adjusting for patient and organization variables, such as ED attendance and use of PHC services at the usual source of care, reflecting a mediating or enhancing effect of patient and other organizational variables on the relationship between female-predominant organizations and these outcome variables.

We controlled for patient characteristics in our analyses. Consequently, the differences observed in case-mix between the 2 types of gender-predominant organization cannot explain outcome differences between the two. Some studies have looked at patient-physician gender dyads to determine optimal combination for increased satisfaction and other care outcomes.^11,16^ They found differences related to the gender of patients and of physicians. We did not replicate these analyses at the organizational level because, though interesting at the individual level, the results would be a bit confusing if applied at the organization level as individual patients could not be matched with individual physicians but only with gender-predominant organizations.

### Limitations

Our study has some limitations. The main one is the lack of information on individual characteristics of physicians and their aggregation at the level of organizations. The original study did not include such information because we did not carry out a survey of individual physicians. Looking at aggregate characteristics of physicians likely misses some variation within organizations that would have been interesting to consider. In this study, the focus was on organizations, not on individual physicians. This constitutes in itself a novel character of our study.

It was possible to link responses of participants to the population survey with their usual source of care but not with their doctors. This limitation adds to the justifications provided earlier for having a dichotomous gender predominance variable rather than a categorical or a continuous one which would be misleading to use in the analysis because we cannot separate out services provided by male and female doctors. Hence, patient experiences of care attributable to male or female doctors in a given organization are primarily related to their relative weight in that organization. Using a dichotomous gender predominance variable partly controlled for that confounding effect

The aggregate information that we had on physicians was obtained through a reliable method in the organization survey. In each organization, we used a reputational approach to identify a key informant who was best informed about the organization. This method can lend itself to possible bias as the opinion of one individual is expected to reflect the viewpoint of the whole organization. Information collected from key informants is generally more complete and more reliable than from individuals who often know little about the organization’s matters and tend to express their personal and limited viewpoint.^40^

Imputation of data to nonrespondent PHC organizations can introduce bias. Based on basic information that we had for all 606 organizations concerning type of PHC organizations (private, community-based, etc), region of location, and size measured by the number of physicians in the setting, we randomly matched a nonrespondent organization to a respondent one that shared the same basic characteristics. This procedure called hot-deck imputation is indicated in that case and likely yields less bias than nonresponse.^26^ In previous publications, we tested the possible magnitude of bias resulting from such imputation. Our sensitivity analyses did not reveal any statistically differences among outcome variables between respondent and nonrespondent PHC organizations.^24,31^

Finally, limiting our study to urban and suburban regions reduces the external validity of our findings and does not enable us to generalize to the other Québec regions.

## Conclusion

Our study is original in that it sheds light on differences between female- and male-predominant types of PHC organizations. Looking at organizations makes the analysis and interpretation of our findings more complex and difficult, but it opens interesting avenues for future research. In many regards, female-predominant PHC organizations are comparable with their male-predominant counterparts, except for accessibility, mainly due to their younger age. Our study clearly stresses the importance of adjusting not only for patient but also organizations’ characteristics and doctors’ age in assessing the impact of female-predominant organizations.

Our findings also reveal potential levers of change for the delivery of PHC services that have not been explored so far. The increased percentage of women in medicine is not simply a demographic phenomenon; it is also a sociological phenomenon. Our study suggests that the increased entry of women in medicine, and particularly in PHC, would likely bring about important changes in PHC organizational settings. Organizational processes through which the influence of female doctors is exerted must be further examined and understood to more accurately assess the future impacts of increased supply of female doctors on the health care system.

## Supplementary Material

Supplementary material

Supplementary material
