# Supplementary material for: Do Gender-Predominant Primary Health Care Organizations Have an Impact on Patient Experience of Care, Use of Services, and Unmet Needs?
Source: Inquiry. 2017 Jun 4;54:0046958017709688. doi: 10.1177/0046958017709688 (PMC5798713; doi:10.1177/0046958017709688)
Supplement: Supplementary material [file Supplementaryfile2_inq-16-0189.pdf]

Supplementary file 2. Experience of care, preventive care, unmet needs and use of services variables

Variables	Coding
**Experience of care* (last two years)**	
**Accessibility of services (score on a 10-point scale)**	
If the doctor who is responsible for your care is not available, you can see another doctor?	**1.**Always; **0.**Never/Sometimes/Often
How long does it take to see the doctor by appointment?	**1.**Less than two weeks; **0.**Two weeks or more
How long does it usually take to get there?	**1.**Less than 15 minutes; **0.**15 minutes or more
The office hours are convenient?	**1.**Strongly agree; **0.**Not at all/A little/Somewhat agree
It is easy to reach someone by telephone to make an appointment?	**1.**Strongly agree; **0.**Not at all/A little/Somewhat agree
It is easy to talk to a doctor or nurse by telephone?	**1.**Strongly agree; **0.**Not at all/A little/Somewhat agree
**Continuity of care (score on a 10-point scale)**	
You see the same doctor?	**1.**Always; **0.**Never/Sometimes/Often
How long have you been going there?	**1.**More than 5 years; **0.**Five years or less
Your medical history is known?	**1.**Strongly agree; **0.**Not at all/A little/Somewhat agree
They are aware of all the prescribed drugs you take?	**1.**Strongly agree; **0.**Not at all/A little/Somewhat agree
You can receive routine ongoing care for a chronic problem?	**1.**Strongly agree; **0.**Not at all/A little/Somewhat agree
**Comprehensiveness (score on a 10-point scale)**	
All your health problems are taken care of whether they are physical or psychological?	**1.**Strongly agree; **0.**Not at all/A little/Somewhat agree
The doctor takes the time to talk to you about prevention and asks you about your lifestyle habits?	**1.**Strongly agree; **0.**Not at all/A little/Somewhat agree
They help you get all the health care services you need?	**1.**Strongly agree; **0.**Not at all/A little/Somewhat agree
Your opinion and what you want are taken into account in the care that you receive?	**1.**Strongly agree; **0.**Not at all/A little/Somewhat agree
You are given help to weigh the pros and cons when you have to make decisions about your health?	**1.**Strongly agree; **0.**Not at all/A little/Somewhat agree
**Responsiveness (score on a 10-point scale)**	
How long do you have to wait between the scheduled time of appointment and the time you actually see the doctor?	**1.**Less than 60 minutes; **0.**60 minutes or more
The staff answers your questions clearly?	**1.**Strongly agree; **0.**Not at all/A little/Somewhat agree
You feel respected?	**1.**Strongly agree; **0.**Not at all/A little/Somewhat agree
You are greeted courteously at the reception?	**1.**Strongly agree; **0.**Not at all/A little/Somewhat agree
Your physical privacy is respected?	**1.**Strongly agree; **0.**Not at all/A little/Somewhat agree
The doctors spend enough time with you?	**1.**Strongly agree; **0.**Not at all/A little/Somewhat agree
The local of the clinic are pleasant?	**1.**Strongly agree; **0.**Not at all/A little/Somewhat agree
**Outcome of care (score on a 10-point scale)**	
The services you get help you to better understand your health problems?	**1.**Strongly agree; **0.**Not at all/A little/Somewhat agree
The services you get help you to prevent certain health problems before they appear?	**1.**Strongly agree; **0.**Not at all/A little/Somewhat agree
The services you get help you to control your health problems?	**1.**Strongly agree; **0.**Not at all/A little/Somewhat agree
The professionals you see encourage you to follow the treatments prescribed?	**1.**Strongly agree; **0.**Not at all/A little/Somewhat agree
The professionals you see help motivate you to adopt good lifestyle habits?	**1.**Strongly agree; **0.**Not at all/A little/Somewhat agree

Supplementary file 2. Continued

**Preventive care* (time specified in guidelines)**	
**Lifestyle habits counselling (score on a 10-point scale)**	
When was the last time someone talked with you about your diet and eating habits? (all)	**1.**In the last 2 years; **0.**Never happened or more than 2 years ago
When was the last time someone talked with you about your physical activity? (all)	**1.**In the last 2 years; **0.**Never happened or more than 2 years ago
When was the last time you were asked whether you smoked? (all)	**1.**In the last 2 years; **0.**Never happened or more than 2 years ago
When was the last time you were recommended to quit smoking? (smokers)	**1.**In the last 2 years; **0.**Never happened or more than 2 years ago
**Cancer and cardiometabolic disorders screening (score on a 10-point scale)**	
When was your last PAP test done? (W, 18-69 years old)	**1.**In the last 3 years; **0.**Never or more than 3 years ago
When was the last time you were advised to pass a mammography? (W, 50-69 years old)	**1.**In the last 2 years; **0.**Never or more than 2 years ago
When was the last time you were prescribed a test to detect blood in your stools (FOBT) and the last time you were advised to pass a colonoscopy? (50-75 years old)	(FOBT) **1.**In the last 2 years; **0.**Never or more than 2 years ago/ (colonoscopy) **1.**In the last 10 years; **0.**Never or more than 10 years ago
When was the last time someone measured your blood pressure? (all)	**1.**2 years ago or less; **0.**Never happened or more than 2 years ago
When was the last time you were prescribed a test to measure your blood sugar level? (40 years old or over)	**1.**In the last 3 years; **0.**Never or more than 3 years ago
When was the last time you were prescribed a blood test to measure your cholesterol level? (W, 50-70; M, 40-70)	**1.**In the last 3 years; **0.**Never or more than 3 years ago
**Unmet needs (last 6 months)**	
Did you feel you needed to see a doctor for a health problem but didn't see one?	**1.**Yes; 0.No
**Utilization of services (last two years)**	
How many times were you hospitalised?	**1.**Once or more; **0.**None
How many times did you go to a hospital emergency room to get medical care?	**1.**Once or more; **0.**None
*How many times did you go to this place to seek care for yourself?**	**1.**6 times or more; **0.**1 to 5 time

* Refer to the experience of care and preventive services at the usual PHC source identified by the respondents
