# Supplementary material for: Do Gender-Predominant Primary Health Care Organizations Have an Impact on Patient Experience of Care, Use of Services, and Unmet Needs?
Source: Inquiry. 2017 Jun 4;54:0046958017709688. doi: 10.1177/0046958017709688 (PMC5798713; doi:10.1177/0046958017709688)
Supplement: Supplementary material [file SupplementaryFile1_inq-16-0189.pdf]

Supplementary file 1. Organizational variables

**Questions**	**Coding**
**Number of physicians in the practice (size)**	
How many general practitioners, including those working part time, currently work at your clinic?	**3.**10 physicians or more; **2.**6-9 physicians; **1.**3-5 physicians; **0.**2 physicians
**Average age of physicians in the practice**	
How many general practitioners at your clinic are…	*Mean*
34 or less?	**3.**60 or more; **2.**57-59; **1.**49-56; **0.**30-48
35 to 49?	
50 to 64?	
65 or more?	
**Time devoted by nurses to follow-up of patients with chronic diseases**	
What are the roles and functions of the nurses in your medical team?	
Follow-up of specific clienteles?	**1.**Yes = At least of these; **0.**No = None of these
Performing clinical activities in the context of standing orders?	
**Time devoted by nurses to counselling on healthy habits**	
What are the roles and functions of the nurses in your medical team?	
Counselling on tobacco use, diet and physical activity?	**1.**Yes = At least of these; **0.**No = None of these
Sexually transmitted and blood borne infections counselling?	
**Use of at least one information technology**	
In your clinic, do you use…	
access to the health and social services telecommunications network?	
a Web-based appointment system for patients?	
computerized tools for continuing professional education?	**1.**Yes = At least of these; **0.**No = None of these
electronic medical records?	
electronic interface to diagnostic imaging laboratory services?	
computerized tools to aid medical decision-making?	
**Collaboration with other PHC practices**	
Does your clinic have formal or informal arrangements with other PHC clinics…	
for planning of services supply?	**1.**Yes = At least of these; **0.**No = None of these
for access to technical services?	
for exchange of resources?	
**Collaboration with hospitals**	
Does your clinic have formal or informal arrangements with hospitals…	
for planning of services supply?	**1.**Yes = At least of these; **0.**No = None of these
for access to technical services?	
for exchange of resources?	
**Services offered on evenings or weekends**	
At your clinic, do you offer…	
services by appointment during weekends?	
services by appointment during weekday evenings (after 6:00 pm)?	**1.**Yes = At least of these; **0.**No = None of these
walk-in services during weekend?	
walk-in services during weekday evenings (after 6:00 pm)?	

Supplementary file 1. Continued

**Prevailing type of visits in the practice**	
What percentage of walk-in visits to all visits do you provide at your clinic?	**2.**Mixed = 26 to 75%;<br>**1.**By-appointment visits = 0% to 25%;<br>**0.**Walk-in visits = 51% or more
**Length of time allowed for each visit**	
At your clinic, how much time is allowed for…<br>*by-appointment consultation?*<br>*emergency consultation?*	**2.**Longest = 21 minutes or more;<br>**1.**Moderate 11 to 20 minutes;<br>**0.**Shortest = 10 minutes or less
**Range of diagnostic and therapeutic services available**	
At your clinic, are the following services available?<br>*Strep-test?*<br>*Skin biopsy?*<br>*IUD insertion?*<br>*Musculo-skeletal injection/aspiration?*<br>*Suture/minor surgery?*<br>*Cervical smear (Pap test)?*<br>*Childhood vaccination?*<br>*Influenza vaccination?*	**2.**Highest = 6 or more of these;<br>**1.**Moderate = 4 or 5 of these;<br>**0.**Lowest = 3 or less of these
**Number of mechanisms available for preventive services delivery**	
At your clinic, do you have…<br>*a reminder system to invite patients to have the recommended screening tests?*<br>*a checklist in the file concerning the preventive clinical practices to carry out with patients, according to current guidelines?*<br>*a tool to support lifestyle habit counselling?*<br>*a reference tool for services offering support for lifestyle changes?*<br>*a chart, in the files of patients with chronic diseases, that includes all the important follow-up components listed in patient management guidelines?*	**2.**Highest = 4 or 5 of these;<br>**1.**Moderate = 2 or 3 of these;<br>**0.**Lowest = 0 or 1 of these
**Number of mechanisms in place for maintaining competency**	
At your clinic, is there anyone who…<br>*organises meetings for case discussions?*<br>*looks after recruitment of physicians and assigns grants practice rights?*<br>*ensures that the quality of medical acts is evaluated?*<br>*organises continuing medical education activities?*	**2.**Highest = 4 of these;<br>**1.**Moderate = 1 to 3 of these;<br>**0.**Lowest = None of these
**Average number of hours physicians devote weekly to clinical activities in the setting**	
How many hours do general practitioners work at your clinics?<br>*Less than 10 hours a week?*<br>*10 to 25 hours a week?*<br>*26 to 40 hours a week?*<br>*More than 40 hours a week?*	*Mean*<br>**2.**More = 33 hours/week or more;<br>**1.**Moderate = 21 to 32 hours/week;<br>**0.**Less = 20 hours/week or less
